# Does participating in community gardens promote sustainable lifestyles in urban settings? Design and protocol of the JArDinS study

**DOI:** 10.1186/s12889-019-6815-0

**Published:** 2019-05-17

**Authors:** Marion Tharrey, Marlène Perignon, Pascale Scheromm, Caroline Mejean, Nicole Darmon

**Affiliations:** 1 MOISA, Univ Montpellier, CIRAD, CIHEAM-IAMM, INRA, Montpellier SupAgro, Montpellier, France; 2 INNOVATION, Univ Montpellier, CIRAD, CIHEAM-IAMM, INRA, Montpellier SupAgro, Montpellier, France

**Keywords:** Diet, Food prices, Environment, Physical activity, Nature, Well-being, Food waste, Natural experiment, Loneliness, Accelerometry

## Abstract

**Background:**

Despite growing evidence for the multiple health benefits of community gardening, longitudinal studies based on quantitative data are needed. Here we describe the protocol of JArDinS, a quasi-experimental study, aimed at assessing the impact of community garden participation (a natural experiment) in the adoption of more sustainable lifestyles.

**Methods:**

Gardeners (*n* = 80) starting gardening in a community garden in Montpellier (France) will be recruited. Volunteers with no experience in community gardening and matched for age range, gender, household income and household composition will be recruited in a control group (*n* = 80). The sustainability of lifestyles in its social/health, environmental and economic dimensions will be assessed from a food supply diary (recording type, quantity and price of foods acquired in a 1-month period and the carbon impact of relevant food trips), a triaxial accelerometer (measuring physical activity) and online questionnaires on mental and social health, sensitivity to food waste, and connection with nature. Change of outcomes after 1 year will be compared between the natural experiment and the control groups.

**Discussion:**

This study will provide information on the impact of participation in a community garden on the different dimensions of sustainability, based on a robust quasi-experimental design allowing causality evaluation.

**Trial registration:**

The JArDinS study was registered at clinicaltrials.gov as NCT03694782. Date of registration: 3rd October 2018, retrospectively registered.

## Background

With 73% of the European population living in urban settlements [1], and an expected increase in this number in the foreseeable future, cities are important determinants of future sustainability and human health [2]. However, urban environment can promote unhealthy lifestyles (such as unhealthy diets and sedentarity) that are known to increase the risk of chronic non-communicable diseases, a major public health challenge today [2]. In particular, exposure to nature and green spaces is known to have a beneficial effect on health and well-being through its impact on physical activity, social contacts, stress and air quality [3, 4]. Hence the presence of green spaces in urban settings is gaining recognition as a way to link ecosystems and human health, and may help achieve more sustainable cities and communities [5, 6].

The recognition of close links between human health and the natural environment is increasingly recognized in public health studies by the use of an ecological approach [7]. Defined by Land and Rayner, the core idea of ‘Ecological Public Health’ is that modern public health, which has so far focused on individuals’ behaviors without considering the ecosystem in which they live, is not sufficient to address today’s health and environmental challenges. The twenty-first century’s public health policies require full consideration of the complex interdependence between people, their health, and their physical and social environments. Recently, Schram-Bijkerk et al. [8] placed urban gardening in the context of urban green space management and valuation and developed a framework illustrating how urban gardening could yield health benefits by interconnecting ecosystem health and human health.

A community garden is a plot of land gardened collectively by a group of people living in an urban area. In addition to the benefit of green space, community gardens offer a place to grow fresh fruit and vegetables, which may help more sustainable food systems to develop. As defined by the High Level Panel of Experts on Food Security and Nutrition, sustainable food systems are those that facilitate healthy, sustainable food practices [9]. Studies of community gardens have shown that gardeners report eating more fruits and vegetables (F&V) than non-gardeners, associated with healthier diets [10–13]. Further, by engaging gardeners in a collective reflection about biodiversity, community gardens can generate a sense of shared personal commitment to sustainability (also called “ecological citizenship”) [14], which could be a driving force for sustainable consumption [15, 16].

However, little is known about the contribution of garden productions to the diet of gardeners. In a previous study that we conducted in poor neighbourhoods of Marseille (France), we found that the household food supply of gardeners contained more F&V than that of their non-gardening neighbours (369 vs. 211 g/d per person, *p* = 0.03) [17]. Unexpectedly, this difference was mostly due to larger purchases of F&V, and not to the garden production. Small sample size, biased control group and cross-sectional design did not allow causal inferences, but these results suggest that community gardens could play a significant role in encouraging healthier dietary practices.

In addition to dietary practices, positive relations between urban gardening and other aspects of human health, such as physical activity, stress reduction and social cohesion, have been observed and summarized in several literature reviews [18–24]. However, most of these studies are restricted to the US. Benefits of community gardens might be different in European cities with diverse social, political and urban contexts. In addition, studies using a longitudinal design with a sufficient sample size are needed to validate emerging hypotheses from qualitative interviews and from cross sectional studies, which still dominate the scientific literature in this field of research [18]. At a time when many European cities are turning their attention to the integration of community gardens on their territory, a more thorough assessment of the impact of community garden participation on gardeners’ lifestyles using experimental design studies is needed. In particular, as pointed out by Alaimo et al. [23], the next generation of research on gardening needs to test causality, namely whether healthier behaviors are due to the effect of gardening as an intervention rather than being a manifestation of prior lifestyles. Natural experiments, in which exposure to the event of interest cannot be practically manipulated by the researcher (such as the community garden participation), provide an opportunity to explore causality in a natural setting [25].

**Fig. 1:**
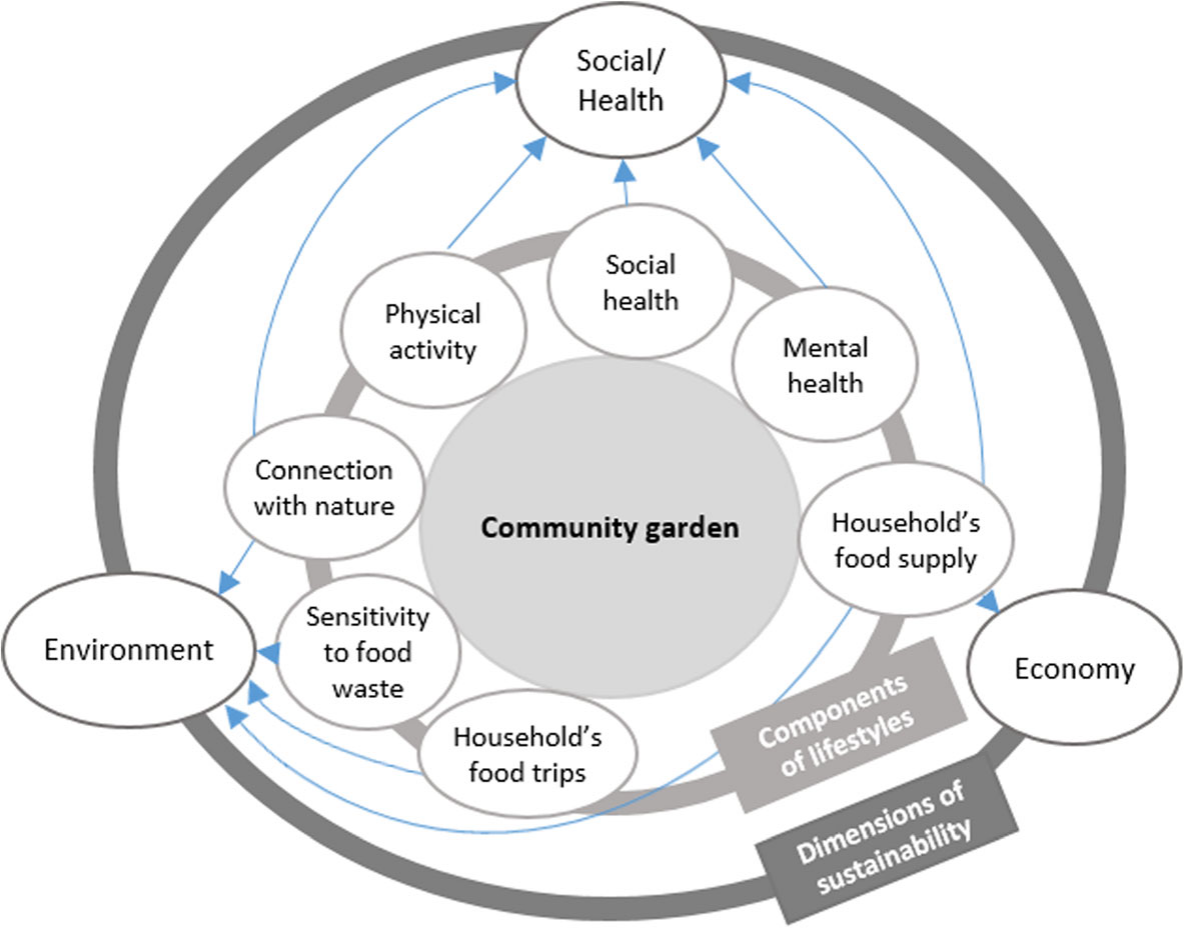
Investigated components of the lifestyles and associated dimensions of sustainability

The JArDinS study is a quasi-experimental research project designed to assess the effect of community garden participation, considered as a natural experiment, on sustainability of gardeners’ lifestyles. Despite the lack of consensus on its definition, the term “lifestyles” is widely used in health promotion, social epidemiology and other branches of public health to mean a cluster of habits that include an individual’s behaviors, inclinations, preferences and values that affect health status [26]. In the present study, the health dimension of gardeners’ lifestyles in relation to their participation in a community garden will be measured in its physical, mental and social components following the World Health Organization’s definition of health [27] by looking at food supply, physical activity, mental well-being and social isolation. An economic and environmental analysis of household food supplies, together with the environmental impact of household food trips, sensitivity to food waste and connection to nature will also be explored jointly with the health dimension to find out to what extent community garden participation can promote more sustainable lifestyles in its social/health, environmental and economic dimensions, the three fundamental pillars of sustainability [28] (Fig. 1). Individual and contextual factors will be also investigated to help understand how they can modulate the impact of community garden participation on outcomes. The present paper describes the protocol of the JArDinS study.

## Methods/design

### Study setting, population and design

The JArDinS study is part of the ongoing SURFOOD-Foodscapes project evaluating the effects of urban foodscape on food styles in the Montpellier Metropole (France). The project is divided into 6 different work packages, the main one consisting of a quantitative survey on food purchase behaviors (Mont’panier survey). The JArDinS study is the work package addressing the impact of urban gardening on sustainable lifestyles.

**Fig. 2:**
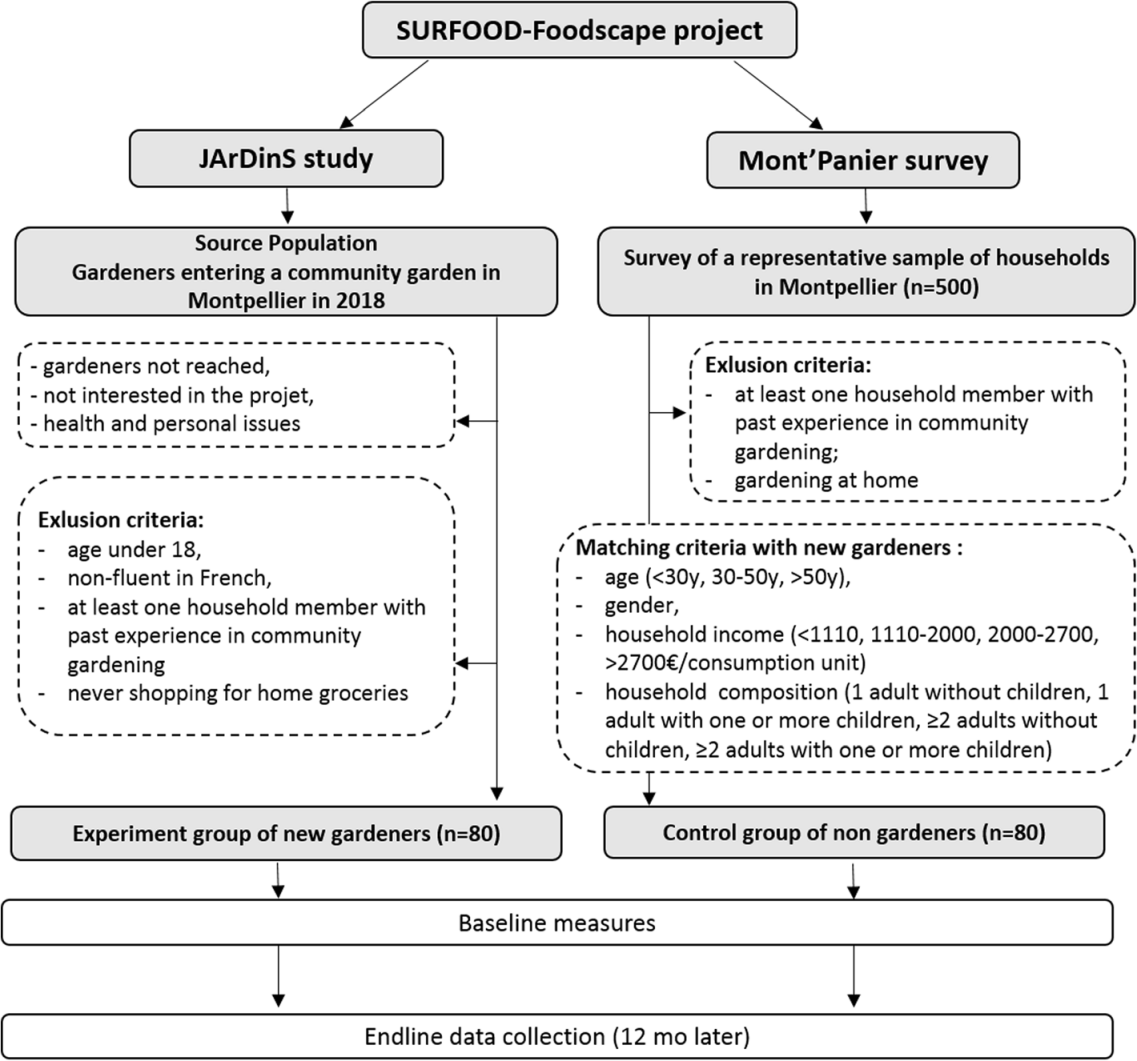
Flow diagram of overall study plan

JArDinS is a pretest-posttest quasi-experimental research project (Fig. 2). The design comprises a natural experiment group of new gardeners starting gardening in a community garden in Montpellier and a control group of participants from a survey on food behaviors undertaken as part of the SURFOOD-Foodscapes project. Participants will be surveyed at enrolment and 12 months later. Quasi-experimental design was chosen rather than a randomized, controlled experiment since random sampling is impossible in French community gardens, where new membership and plot renewal in community gardens are decided by local authorities or private managers.

Baseline sample size was estimated based on a priori power analysis considering household supply of F&V as the primary outcome. The hypothesis was derived from our knowledge of the food supply content of community gardeners vs. non-gardeners, based on data collected in a cross-sectional study previously conducted in the south of France [17]. Assuming a potential attrition rate of 30% and a correlation of 0.6 across repeated measures, a total of 160 participants is required to detect a 30% increase in F&V supply in the new gardeners group (namely one portion of F&V) for 80% power at the 5% alpha level.

The recruitment of new gardeners (natural experiment group) is taking place in 2018 throughout the gardening season (from March to November). A close working relationship between the research team and the local organizations in charge of organizing and managing community gardens in Montpellier is facilitating the recruitment process. When new members join a garden, the leader informs the research team, who come to the garden and introduce the study to all the potential participants. A home visit is then be scheduled for those interested in the study, during which further explanation of requirements, data collection procedures, security and confidentiality of the information gathered is given.

The inclusion criteria for new gardeners are: starting gardening in a community garden, willingness to be involved in the study for 1 year, age above 18 years, ability to read French, and residence in the city of Montpellier. The exclusion criteria are: past experience of at least one household member in community gardening, and never shopping for home groceries.

Data collected from new gardeners will be compared with those from a group of residents who have never participated in a community garden. This control group will be selected using frequency matching from a subsample of persons who participate in a survey on food behaviors undertaken as part of the SURFOOD-Foodscapes project (“Mont’Panier” survey), and who have no experience in community or home gardening. This survey is being conducted in 500 households of Montpellier from May to December 2018 using quota sampling based on age and household composition. Frequency matching involves the selection of an entire stratum of reference subjects (control group) with matching-factor values equal to that of a stratum of index subjects (natural experiment group) [29]. The control group will be matched for age range, gender, household income and household composition, and live as close as possible to the new gardeners.

### Data collection steps

A first home visit will be scheduled to provide participants with data collection materials (a food supply diary, a hip-worn triaxial accelerometer and an online questionnaire), together with instructions on how to use it. A second and third home visit will be set up approximately 10 days later to collect the accelerometer and check that participants are filling out the food supply diary properly, and 1 month later to retrieve the food supply diary. The interviewer will be reachable at all times during data collection to respond to any concerns.

For the control group, the food supply diary will be completed as part of the “Mont’Panier” survey, with the possibility of receiving it in printed form or online. For the accelerometer and the questionnaire, home visits will be scheduled according to the same protocol as for the experiment group.

Data will be collected at baseline (*t*_0_) and exactly 12 months later (*t*_1_). Participants will receive a 15 € voucher at *t*_0_ and at *t*_1_ for returning all data collection materials duly completed.

### Lifestyles sustainability assessment

The effect of community garden participation on gardeners’ lifestyles sustainability will be investigated by looking at specific components of the lifestyles in its social/health, environment and economic dimensions. All the outcome variables are summarized in Table 1.

**Table 1:** Outcome variables of the JArDinS study

Variable	Level	Data collection tools	Measurement method
Main outcome measures : lifestyles sustainability			
Health/Social dimension			
Healthiness of household’s food supply	Household	Food supply diary	Share of major food groups, especially F&V in household supply; indicators of nutritional quality: Mean Adequacy Ratio (MAR), Mean Excess Ratio (MER)
Physical activity: PAEE and total EE	Individual	Triaxial accelerometer (Actigraph)	Automatic activity-recognition (AAR) algorithm with an activity-specific count-based model
Mental health	Individual	Online questionnaire	Warwick-Edinburgh Mental Well-being Scale (WEMWBS): 14 items on a 5-point Likert scale.
Social health	Individual	Online questionnaire	UCLA Loneliness Scale (Version 3): 20 items on a 4-point Likert scale.
Environmental dimension			
Environmental impact of household’s food supply	Household	Food supply diary	GHGE (in g CO_2_eq), atmospheric acidification (in g SO_2_eq) and marine eutrophication (in g Neq)
Environmental impact of household’s food trip	Household	Food supply diary	GHGE (in g CO_2_eq) of food trip
Sensitivity to food waste	Individual	Online questionnaire	Sensitivity to food waste scale: 8 items on a 7-point Likert scale
Connection with nature	Individual	Online questionnaire	Nature Relatedness Scale: 21 items on a 5-point Likert scale
Economic dimension			
Cost of food supply	Household	Food supply diary	Total food household food expenditure and share of each food group and subgroup
Other measures :			
Sociodemographic variables	Household Individual	Online questionnaire	Composition, income, perceived financial situation age, sex, education, employment of each member
Away-from-home consumption	Individual	Online questionnaire	2 questions evaluating the frequency of away-from-home consumption, and food groups mainly consumed away from home.
Experience in gardening	Individual	Online questionnaire	2 questions about previous experience in gardening and perceived competence. (ranked as ‘beginner’ ‘intermediate’ and ‘advanced’)

#### Social/Health dimension

Physical, mental and social health behaviors will be assessed by looking at healthiness of food supply, physical activity, mental well-being and social isolation.

***Healthiness of food supply*** To assess household food supply, each participant will be issued with a food supply diary, in which they will be asked to record details of their household food supply and relevant trips over a 1-month period. Step-by-step instructions on how to complete the food supply diary will be given to participants and also specified at the front of the diary. Household food supply includes food purchases, food gifts/donations, and any crops from the garden. Away-from-home food consumption will not be recorded. For each food purchase, participants will be asked to provide details of date, place of purchase, foods purchased (name, quantity and expense incurred). When grocery and supermarket till receipts are available, participants will be asked to collect them in an envelope at the back of the food supply diary. To facilitate data entry, all family members will be encouraged to help fill out the diary. All food items purchased will be classified into 9 groups and 29 subgroups to see the share of major food groups in the household supply. The nutritional composition of each food item will be determined using the national food composition database and completed by recent market foods and recipes. Two indicators of nutritional quality previously described by Vieux et al. will be used to estimate the nutritional quality of 2000 kcal of food supply: the mean adequacy ratio (MAR) and the mean excess ratio (MER) [30]. The MAR is an indicator of good nutritional quality and represents the mean percentage of daily recommended intakes for 20 key nutrients. The MER is an indicator of bad nutritional quality and represents the mean daily percentage of maximum recommended values for three unhealthy nutrients, namely saturated fatty acids, sodium, and free sugars (defined by the WHO as added sugars plus sugars naturally present in honey, syrups, and fruit juices) [31].

#### Physical activity

Physical activity (PA) will be measured by triaxial accelerometry using an Actigraph activity monitor (wGT3X-BT or wActiSleep-BT, Actigraph, Pensacola, FL, USA). The Actigraph is a motion sensor that measure acceleration from body movements in three orthogonal planes (anteroposterior, mediolateral, and vertical) [32]. The Actigraph will be set to collect data at a sampling frequency of 30 Hz. Participants will be instructed to wear the Actigraph fitted with an elastic strap on the right side of the hip for 9 consecutive days, except for bathing and performing activities in water. The Actigraph is accompanied by an activity logbook in which participants will be required to record time awake and sleeping daily, and time and duration of any strenuous exercise or device removal. Physical activity energy expenditure will be estimated directly from raw triaxial accelerometry data using a previously validated model that combines an automatic activity-recognition algorithm with an activity-specific count-based model [33]. The automatic activity-recognition algorithm relies on four signal features from time and frequency domains to classify 6 s consecutive time spans into six posture/activity categories: a) lying down, b) slouching, c) sitting, d) standing still, e) moving on foot (sweeping, treading, walking, running, etc.), and f) cycling [34]. In addition, usual physical activity in four domains (leisure, work, commuting, home) during the past month will also be assessed with an online questionnaire using the physical activity Recent Physical Activity Questionnaire (RPAQ). Online RPAQ has been found to provide a good estimate of moderate-to-vigorous physical activity in ten European countries including France [35].

***Mental health*** Mental well-being will be assessed using the Warwick-Edinburgh Mental Well-Being Scale (WEMWBS) using an online questionnaire [36]. WEMWBS rates 14 items on a 5-point Likert scale, in which all items are worded positively and address aspects of positive mental health. Total scores range from 14 to 70, with higher scores indicating a higher level of mental well-being. WEMWBS has been validated in French general populations [37].

***Social health*** Perceived social isolation or loneliness can impair physical and mental health by influencing psychological processes that alter physiological functioning, reduce sleep quality, and increase morbidity and mortality [38]. The UCLA Loneliness Scale (UCLA-3) will be used as a common measure of subjective feelings of loneliness and of social isolation [39]*.* UCLA-3 rates 20 items (11 positive and 9 negative) on a 4-point Likert scale. Total scores range from 20 to 70, with higher scores for stronger feelings of loneliness*.*

#### Environmental dimension

The environmental dimension of lifestyles will be assessed through the environmental impact of the household’s food supply and food trips, and sensitivity to food waste. Connection with the natural environment will also be assessed, since community gardens offer a place of contact with nature and reflection about biodiversity, which in turn could foster environment-friendly practices.

***Environmental impact of household’s food supplies*** Greenhouse gas emissions (GHGE, in grams of carbon dioxide equivalents, g CO_2_eq), atmospheric acidification (in grams of sulphur dioxide equivalents, g SO_2_eq) and marine eutrophication (in grams of nitrogen equivalents, g Neq) related to the household’s food supply will be computed using estimates from the French ‘SUStable’ table [40]. This database provides estimates of the three indicators per gram of food for 212 commonly consumed generic foods*,* based on a hybrid method combining input/output and LCA approaches, as previously described by Bertoluci et al. [41]. Estimates will be allocated to each food collected in the food supply diary by linking them to the closest food in the ‘SUStable’ database.

***Environmental impact of household food trips*** For each food purchase recorded in the food diary supply, participants will provide details of the trip made (origin/where the trip started, destination/where the trip ended, and mode of transport). All locations will be geocoded using QGIS 2.18 to compute the total distance travelled for each food trip. Information collected on the origin/destination of each trip will describe the overall travel pattern associated with the food trips and tell whether food trips are integrated into multiple-purpose trip. The GHGE related to food trips will then be calculated using the French government’s methodology (decree No. 2011–1336 of October 24, 2011) to estimate carbon dioxide emissions (g/km) in the transport sector. Briefly, the distance travelled for food purchase will be computed depending on the mode of transport (walking, cycling, car, motorbike, tram and bus). For multi-purpose trips, the distance specifically travelled for food purchase will be estimated as the additional distance travelled to go to the point of food purchase (estimated as the difference between the total distance travelled during the trip and the distance travelled during the same trip without going to the point of food purchase). For each food trip, the distance travelled specifically for food purchase will be multiplied by the energy source consumption of the transport used, and by the corresponding GHGE factor of source consumption. For each household, GHGE related to food trips will be calculated as the sum of all food trips during the period of data collection.

***Sensitivity to food waste*** We will assess how much importance participants attach to waste and how emotionally affected they are by it, using a previously published set of 8 items rated on a 7-point Likert scale [42]. Because the topic of food waste is likely to suffer from a social desirability response bias, a widely used social desirability scale – the Balanced Inventory of Desirable Responding – in its short version was added to the questionnaire [43].

***Connection with nature*** Affective, cognitive, and experiential aspects of individuals’ connection to nature will be assessed using the validated Nature Relatedness scale, with 21 items rated on a 5-point Likert scale [44].

#### Economic dimension

Monthly household food expenditure and the contribution of each food group and subgroup to total food expenditure will be estimated using food expenses data collected in the food supply diary. We will assess whether a change in the healthiness of food supply influences the level of household food expenditure.

### Other measures

#### Socioeconomic and demographic variables

Socioeconomic and demographic characteristics of participants will be assessed by online questionnaire and include household composition, incomes and how they perceive their financial situation, age, gender and education level of each household member.

#### Away-from-home consumption

Away-from-home consumption of each household member will also be estimated, since a high away-from-home food consumption can impair the ability of the food supply to provide an accurate estimation of dietary quality and nutrient intakes [45]. To this end, participants will be asked by questionnaire how often they generally eat away from home (four categories possible: company restaurant/canteen, restaurant and catering, fast-food, meal with friends, with six possible answers (“every day”, “4–6 times/wk”, “1–3 times/wk”, “1–3 times/month”, “< 1 time/month”, “never or rarely”). Household members will be also asked whether they tend to consume F&V, meat, fish and dairy products mostly at home, away-from-home, both in and outside the home, or never.

#### Gardening experience

Gardening experience prior to the project will be assessed by an online questionnaire asking participants about their experiences and perceived competence in gardening.

### Planned statistical analysis

Summary statistics (mean, median, standard deviation and frequency distribution) will be generated for baseline characteristics. Baseline characteristics will be compared between the experiment and control groups using one-way ANOVA or a Kruskal-Wallis test for continuous variables, and chi-square or Fisher’s exact tests, for categorical variables. The magnitude of change within and between groups between *t*_0_ and *t*_1_ will be calculated.

Data will be analysed to address the research questions, applying appropriate linear mixed models. Differences within and between groups in the outcome parameters will be analysed. Time, group and their interaction will be defined as fixed factors. Subject and group will be included as random factors. If imbalances occur between groups, the baseline values will be treated as covariates.

All analyses will be performed with the SAS statistical software package Ver. 9.4 for Windows (SAS Institute, Cary, NC, USA), with statistical significance at *p* < 0.05.

## Discussion

There is growing interest in urban gardens as a way to address environmental and health issues of urbanization [8]. The JArDinS study will lend insight into the effectiveness of community gardens as a tool for promoting sustainable health strategies in a European context. In the field of public health, strategies that promote a sustainable lifestyles are needed to respond to current and future problems and challenges. We will therefore go beyond the health components of the lifestyles and add environmental and economic considerations. For the health dimension, we argue that after 1 year, the experience of the community garden should lead to a heathier food supply, increased physical activity and a feeling of more mental well-being and less social isolation. For the environmental dimension, we will examine to what extent the environmental awareness generated by the experience of the community garden can foster a closer connection with nature, more sustainable food supply strategies and greater sensitivity to food waste. For the economic dimension, we will determine whether the improvement in the healthiness of food supply impacts the level of household food expenditure after 1 year of community gardening. The components of the lifestyles will be compared with one another to help gain an overall understanding of the lifestyles. In particular, the compatibility between the three dimensions of sustainability will be explored for the food supply by looking at the relationships between nutritional quality indicators, cost and the environmental impact of household food supply, together with the carbon impact of trips made for food purchases.

A major strength of this study is its longitudinal design with the use of pretest and posttest assignment and a control group that will explore the causality inference. Another strength is the objective measurement of food supply practices and physical activity using the food supply diary and the Actigraph. Compared to self-report methods, objective measurement tools are insensitive to recall and response bias and so provide more relevant and reliable information [46–49]. In earlier literature, the relation between gardening and nutrition was mostly assessed through declared F&V intake [10–13]. At the interface between food environment and food consumption, food supply has the advantage of providing unique in-depth information on food sources and food items along with accurate up-to-date information on expenditures and quantities purchased [50, 51]. Similarly, accelerometers have gained considerable popularity in recent years as an accurate tool to measure energy expenditure and estimate physical activity. Accelerometers are convenient and non-invasive and can be used relatively easily in free-living conditions to capture large amounts of data over several days [49]. Besides, the automatic activity-recognition model used in the study to derive information on physical activity from raw triaxial accelerometry data was found to improve physical activity energy expenditure and total energy expenditure predictions from a hip-worn triaxial-accelerometer in free-living conditions, compared with traditional count-based prediction approaches [33].

The study has some limitations. First, although several well-known confounders will be measured in the study and controlled in the analyses, the use of a natural experiment makes it difficult to control all extraneous variables, and so unknown and unmeasured extraneous variables may affect the results, threatening validity. Second, the natural experiment and control groups might differ in some pre-study characteristics because participants are not assigned randomly to the two groups, which is liable to bias the estimates of the experiment. Non-random design can threaten the internal validity of the study, which is critical for determining a causal relationship [25]. To ensure similarity between groups, the members of the control group will be selected using pairwise matching on sociodemographic characteristics known to be associated with the outcomes of interest. Third, we will collect frequency and type of foods consumed away from home, but this information lacks precision regarding the quantity consumed and the price paid for food out of home. Nonetheless, studies have shown that nutrients derived from foods purchased at supermarkets provide a good estimate of dietary intake [51–53]. In addition, in France, most meals are still consumed at home [54] and so we expect food supply to be a good proxy of food intake. Fourth, the economic dimension of sustainability will be assessed only by the cost of the diet: other aspects such as ethical consideration (e.g., economic fairness including fair remuneration for producers) [55] will not be investigated.

Public policies are now emerging in France to promote good health and sustainable development of cities. For instance, community gardening undertaken jointly with other food initiatives (at local and national levels) such as Community Supported Agriculture, or projects at canteens could favour access for all to healthy, good quality local products and thus contribute to more sustainable food systems. The JArDinS study based on a natural experiment will yield findings to guide public and private policies for the organization, physical planning and assignment of urban areas. If the study finds evidence for potential benefits of community gardens to attract individuals to more sustainable lifestyles, then access to a community garden, as way to help promote health and wellness, needs to be facilitated.

## Abbreviations

F&V: Fruit and vegetables
GHGE: Greenhouse gas emissions
MAR: Mean adequacy ratio
MER: Mean excess ratio
PA: Physical activity
RPAQ: Recent physical activity questionnaire RPAQ
WEMWBS: Warwick-Edinburgh Mental Well-Being Scale
WHO: World Health Organization
wk.: Week

## References

[1] Nabielek K, Hamers D, Evers D (2016). Cities in Europe - facts and figures on cities and urban area.

[2] Moore M, Gould P, Keary BS (2003). Global urbanization and impact on health. Int J Hyg Environ Health.

[3] Pretty J, Barton J, Colbeck I, Hine R, Mourato S, Mackerron G (2011). Health Values from Ecosystems. Human Well-being | Chapter 23.

[4] Hartig T, Mitchell R, de Vries S, Frumkin H (2014). Nature and health. Annu Rev Public Health.

[5] Tzoulas K, Korpela K, Venn S, Yli-Pelkonen V, Kaźmierczak A, Niemela J, James P (2007). Promoting ecosystem and human health in urban areas using green infrastructure: a literature review. Landsc Urban Plan.

[6] James P, Tzoulas K, Adams MD, Barber A, Box J, Breuste J, Elmqvist T, Frith M, Gordon C, Greening KL, Handley J, Haworth S, Kazmierczak AE, Johnston M, Korpela K, Moretti M, Niemelä J, Pauleit S, Roe MH, Sadler JP, Ward Thompson C (2009). Towards an integrated understanding of green space in the European built environment. Urban For Urban Green.

[7] Lang T, Rayner G. Ecological public health: the 21st century's big idea? An essay by Tim Lang and Geof Rayner. BMJ. 2012;345:e5466. 10.1136/bmj.e5466 22915666

[8] Schram-Bijkerk D, Otte P, Dirven L, Breure AM (2018). Indicators to support healthy urban gardening in urban management. Sci Total Environ.

[9] HLPE Nutrition and food systems (2017). A report by the High Level Panel of Experts on Food Security and Nutrition of the Committee on World Food Security; Rome.

[10] Alaimo K, Packnett E, Miles RA, Kruger DJ (2008). Fruit and vegetable intake among urban community gardeners. J Nutr Educ Behav.

[11] Litt JS, Soobader M-J, Turbin MS, Hale JW, Buchenau M, Marshall JA (2011). The influence of social involvement, neighborhood aesthetics, and community garden participation on fruit and vegetable consumption. Am J Public Health.

[12] Barnidge EK, Hipp PR, Estlund A, Duggan K, Barnhart KJ, Brownson RC (2013). Association between community garden participation and fruit and vegetable consumption in rural Missouri. Int J Behav Nutr Phys Act.

[13] Litt JS, Schmiege SJ, Hale JW, Buchenau M, Sancar F (2015). Exploring ecological, emotional and social levers of self-rated health for urban gardeners and non-gardeners: a path analysis. Soc Sci Med.

[14] Dobson A (2003). Citizenship and the environment.

[15] Seyfang G (2006). Ecological citizenship and sustainable consumption: examining local organic food networks. J Rural Stud.

[16] Asvatourian V, Craig T, Horgan GW, Kyle J, Macdiarmid JI (2018). Relationship between pro-environmental attitudes and behaviour and dietary intake patterns. Sustain Prod Consum.

[17] Martin P, Consalès J-N, Scheromm P, Marchand P, Ghestem F, Darmon N (2017). Community gardening in poor neighborhoods in France: a way to re-think food practices?. Appetite.

[18] Draper C, Freedman D (2010). Review and analysis of the benefits, purposes, and motivations associated with community gardening in the United States. J Community Pract.

[19] McCormack LA, Laska MN, Larson NI, Story M (2010). Review of the nutritional implications of farmers’ markets and community gardens: a call for evaluation and research efforts. J Am Diet Assoc.

[20] Guitart D, Pickering C, Byrne J (2012). Past results and future directions in urban community gardens research. Urban For Urban Green.

[21] Genter C, Roberts A, Richardson J, Sheaff M (2015). The contribution of allotment gardening to health and wellbeing: a systematic review of the literature. Br J Occup Ther.

[22] Egli V, Oliver M, Tautolo E-S (2016). The development of a model of community garden benefits to wellbeing. Prev Med Rep.

[23] Alaimo K, Beavers AW, Crawford C, Snyder EH, Litt JS (2016). Amplifying health through community gardens: a framework for advancing multicomponent, behaviorally based neighborhood interventions. Curr Environ Health Rep.

[24] Al-Delaimy WK, Webb M (2017). Community gardens as environmental health interventions: benefits versus potential risks. Curr Environ Health Rep.

[25] Shadish WR, Cook TD, Campbell DT. Experimental and quasi-experimental designs for generalized causal inference: Wadsworth Cengage Learning; 2002.

[26] Frohlich KL, Corin E, Potvin L (2001). A theoretical proposal for the relationship between context and disease. Sociol Health Illn.

[27] World Health Organization (1948). Preamble to the Constitution of the World Health Organization as adopted by the International Health Conference, New York, 19-22 June, 1946.

[28] Brundtland GH. Report of the world commission on environment and development: our common future. p. 1897.

[29] Rothman KJ, Greenland S (1998). Modern epidemiology.

[30] Vieux F, Soler L-G, Touazi D, Darmon N (2013). High nutritional quality is not associated with low greenhouse gas emissions in self-selected diets of French adults. Am J Clin Nutr.

[31] Joint WHO/FAO Expert Consultation Diet, nutrition, and the prevention of chronic diseases; World Heal.; 2003.

[32] Chen KY, Bassett DR (2005). The technology of accelerometry-based activity monitors: current and future. Med Sci Sports Exerc.

[33] Garnotel, M.; Bastian, T.; Romero-Ugalde, H.-M.; Maire, A.; Dugas, J.; Zahariev, A.; Doron, M.; Jallon, P.; Charpentier, G.; Franc, S.; Blanc, S.; Bonnet, S.; Simon, C. Prior automatic posture and activity identification improves physical activity energy expenditure prediction from hip-worn triaxial Accelerometry. J Appl Physiol 2017, japplphysiol00556.2017. 10.1152/japplphysiol.00556.2017 29191980

[34] Bastian T, Maire A, Dugas J, Ataya A, Villars C, Gris F, Perrin E, Caritu Y, Doron M, Blanc S, Jallon P, Simon C (2015). Automatic identification of physical activity types and sedentary behaviors from triaxial accelerometer: laboratory-based calibrations are not enough. J Appl Physiol.

[35] Golubic R, May AM, Benjaminsen Borch K, Overvad K, Charles M-A, Diaz MJT, Amiano P, Palli D, Valanou E, Vigl M, Franks PW, Wareham N, Ekelund U, Brage S (2014). Validity of electronically administered recent physical activity questionnaire (RPAQ) in ten European countries. PLoS One.

[36] Tennant R, Hiller L, Fishwick R, Platt S, Joseph S, Weich S, Parkinson J, Secker J, Stewart-Brown S (2007). The Warwick-Edinburgh mental well-being scale (WEMWBS): development and UK validation. Health Qual Life Outcomes.

[37] Trousselard M, Steiler D, Dutheil F, Claverie D, Canini F, Fenouillet F, Naughton G, Stewart-Brown S, Franck N (2016). Validation of the Warwick-Edinburgh mental well-being scale (WEMWBS) in French psychiatric and general populations. Psychiatry Res.

[38] Hawkley LC, Cacioppo JT (2010). Loneliness matters: a theoretical and empirical review of consequences and mechanisms. Ann Behav Med.

[39] Russell DW (1996). UCLA loneliness scale (version 3): reliability, validity, and factor structure. J Pers Assess.

[40] Gazan R, Barré T, Perignon M, Maillot M, Darmon N, Vieux F (2018). A methodology to compile food metrics related to diet sustainability into a single food database: application to the French case. Food Chem.

[41] Bertoluci G, Masset G, Gomy C, Mottet J, Darmon N (2016). How to build a standardized country-specific environmental food database for nutritional epidemiology studies. PLoS One.

[42] Le Borgne G, Sirieix L, Costa S (2018). Perceived probability of food waste: influence on consumer attitudes towards and choice of sales promotions. J Retail Consum Serv.

[43] Hart CM, Ritchie TD, Hepper EG, Gebauer JE (2015). The balanced inventory of desirable responding short form (BIDR-16). SAGE Open.

[44] Nisbet EK, Zelenski JM, Murphy SA (2009). The nature relatedness scale. Environ Behav.

[45] Appelhans BM, French SA, Tangney CC, Powell LM, Wang Y (2017). To what extent do food purchases reflect shoppers’ diet quality and nutrient intake?. Int J Behav Nutr Phys Act.

[46] Prince SA, Adamo KB, Hamel M, Hardt J, Connor Gorber S, Tremblay M (2008). A comparison of direct versus self-report measures for assessing physical activity in adults: a systematic review. Int J Behav Nutr Phys Act.

[47] Sylvia LG, Bernstein EE, Hubbard JL, Keating L, Anderson EJ (2014). Practical guide to measuring physical activity. J Acad Nutr Diet.

[48] Dowd KP, Szeklicki R, Minetto MA, Murphy MH, Polito A, Ghigo E, van der Ploeg H, Ekelund U, Maciaszek J, Stemplewski R, Tomczak M, Donnelly AE (2018). A systematic literature review of reviews on techniques for physical activity measurement in adults: a DEDIPAC study. Int J Behav Nutr Phys Act.

[49] Hills AP, Mokhtar N, Byrne NM (2014). Assessment of physical activity and energy expenditure: an overview of objective measures. Front Nutr.

[50] French SA, Shimotsu ST, Wall M, Gerlach AF (2008). Capturing the Spectrum of household food and beverage purchasing behavior: a review. J Am Diet Assoc.

[51] Ransley JK, Donnelly JK, Khara TN, Botham H, Arnot H, Greenwood DC, Cade JE (2001). The use of supermarket till receipts to determine the fat and energy intake in a UK population. Public Health Nutr.

[52] Ransley JK, Donnelly JK, Botham H, Khara TN, Greenwood DC, Cade JE (2003). Use of supermarket receipts to estimate energy and fat content of food purchased by lean and overweight families. Appetite.

[53] Eyles H, Jiang Y, Ni Mhurchu C (2010). Use of household supermarket sales data to estimate nutrient intakes: a comparison with repeat 24-hour dietary recalls. J Am Diet Assoc.

[54] Volatier JL. Enquête INCA (Individuelle et Nationale sur les Consommations Alimentaires). AFSSA, Agence Française de Sécurité Sanitaire des Aliments, Editor. Lavoisier, Paris, 2000, p. 158.

[55] Johnston JL, Fanzo JC, Cogill B (2014). Understanding sustainable diets: a descriptive analysis of the determinants and processes that influence diets and their impact on health, food security, and environmental sustainability. Adv Nutr.

